# Endoplasmic Reticulum Stress Cooperates in Zearalenone-Induced Cell Death of RAW 264.7 Macrophages

**DOI:** 10.3390/ijms160819780

**Published:** 2015-08-20

**Authors:** Fenglei Chen, Qian Li, Zhe Zhang, Pengfei Lin, Lanjie Lei, Aihua Wang, Yaping Jin

**Affiliations:** 1 Key Laboratory of Animal Biotechnology of the Ministry of Agriculture, Northwest Agriculture and Forestry University, Yangling 712100, Shaanxi, China; E-Mails: cfl0604114114@163.com (F.C.); liqian364@163.com (Q.L.); pipixiaoguai0118@163.com (Z.Z.); linpengfei@nwsuaf.edu.cn (P.L.); 2 College of Veterinary Medicine, Northwest Agriculture and Forestry University, Yangling 712100, Shaanxi, China; E-Mails: leilanjie1988@126.com (L.L.); aihuawang1966@163.com (A.W.)

**Keywords:** zearalenone, endoplasmic reticulum stress, *CHOP*, RAW 264.7 macrophages

## Abstract

Zearalenone (ZEA) is a fungal mycotoxin that causes cell apoptosis and necrosis. However, little is known about the molecular mechanisms of ZEA toxicity. The objective of this study was to explore the effects of ZEA on the proliferation and apoptosis of RAW 264.7 macrophages and to uncover the signaling pathway underlying the cytotoxicity of ZEA in RAW 264.7 macrophages. This study demonstrates that the endoplasmic reticulum (ER) stress pathway cooperated in ZEA-induced cell death of the RAW 264.7 macrophages. Our results show that ZEA treatment reduced the viability of RAW 264.7 macrophages in a dose- and time-dependent manner as shown by the 3-[4,5-dimethylthiazol-2-yl]-2,5-diphenyltetrazolium bromide assay (MTT) and flow cytometry assay. Western blots analysis revealed that ZEA increased the expression of glucose-regulated protein 78 (*GRP78*) and CCAAT/enhancer binding protein homologous protein (*CHOP*), two ER stress-related marker genes. Furthermore, treating the cells with the ER stress inhibitors 4-phenylbutyrate (4-PBA) or knocking down *CHOP*, using lentivirus encoded short hairpin interfering RNAs (shRNAs), significantly diminished the ZEA-induced increases in GRP78 and CHOP, and cell death. In summary, our results suggest that ZEA induces the apoptosis and necrosis of RAW 264.7 macrophages in a dose- and time-dependent manner via the ER stress pathway in which the activation of *CHOP* plays a critical role.

## 1. Introduction

Zearalenone (ZEA) is a non-steroidal estrogenic fungal mycotoxin produced by *Fusarium* that is commonly found in the cereal grain used for animal and human food [1,2]. ZEA possesses estrogenic activity and produces reproductive disorders in farm animals [3,4,5,6]. In addition to its estrogenic activity, ZEA has been believed to be immunotoxic [7,8,9,10,11,12], hepatotoxic [13,14,15] and genotoxic [15,16]. ZEA has been shown to have various actions in different types of cells. In Vero, Caco-2 and DOK cells, ZEA has been demonstrated to produce DNA fragmentation, apoptosis and alteration in cell cycle progression [17]. In human hepatocytes (HepG2), ZEA causes oxidative DNA damage and glutathione depletion via the p53-dependent mitochondrial signaling pathway [17,18]. However, in RAW 264.7 macrophages, ZEA induces apoptosis and necrosis in RAW 264.7 macrophages via the apoptosis-inducing factor (AIF) and reactive oxygen species (ROS)-mediated signaling pathways, and this process was found to be related to the activation of p53 and Jun-N-terminal kinase (JNK)/p38 [19,20]. p53 and JNK/p38 also activates endoplasmic reticulum (ER) stress-related genes, such as CCAAT/enhancer binding protein homologous protein (*CHOP*) [21,22,23,24]. Furthermore, ZEA has been previously reported to induce ER stress in other cells. We therefore were interested to investigate whether ZEA may induce ER stress in RAW 264.7 macrophages.

One of the major physiological functions of the endoplasmic reticulum is to modify, process and fold secretory and membrane proteins. However, the accumulation of misfolded or unfolded proteins induces ER stress. To restore ER function, eukaryotic cells normally respond via transcriptional induction, translational attenuation and ER-related degradation (ERAD), which results from the ER stress response [25]. The ER stress response activates gene transcription of molecular chaperones in the ER, such as glucose-regulated protein 78 (*GRP78*) and *CHOP*. *GRP78* shows a marked up-regulation in ER chaperones in response to ER stress [26]. GRP78 binds to inositol-requiring enzyme 1α (IRE1α), double-stranded RNA-dependent protein kinase (PKR)-like ER kinase (PERK), and activating transcription factor (ATF)-6 in homeostasis, all of which are ER-localized protein sensors for ER stress. When these sensors recognize more significant stress, GRP78 separates from these sensors and interacts with misfolded or unfolded proteins to restore homeostasis [27]. However, severe or prolonged ER stress will trigger cell death. Cell death proceeds through obvious pathways including the activation of CHOP, JNK and caspase-12. *CHOP*, an apoptotic transcriptional factor induced in response to ER stress, is also a popular marker for the assessment of ER stress [28].

Banjerdpongchai found that ZEA induces apoptosis in human leukemic cells via ER stress signaling, in which the endoplasmic reticulum protein 29 (*ERp29*) mRNA transcript levels increased [29]. Recently, our results showed that ZEA induced apoptosis in mouse Leydig cells via ER stress signaling, in which knockdown of *GRP78* diminished ZEA-induced apoptosis [30]. However, the involvement of ER stress in ZEA-induced apoptosis in macrophages remains unknown. The present study aims to detect the effect of ZEA on cell death in macrophages and to examine whether this process is related to ER stress.

## 2. Results

### 2.1. Effect of ZEA on the Proliferation and Apoptosis of RAW 264.7 Macrophages

**Figure 1:**
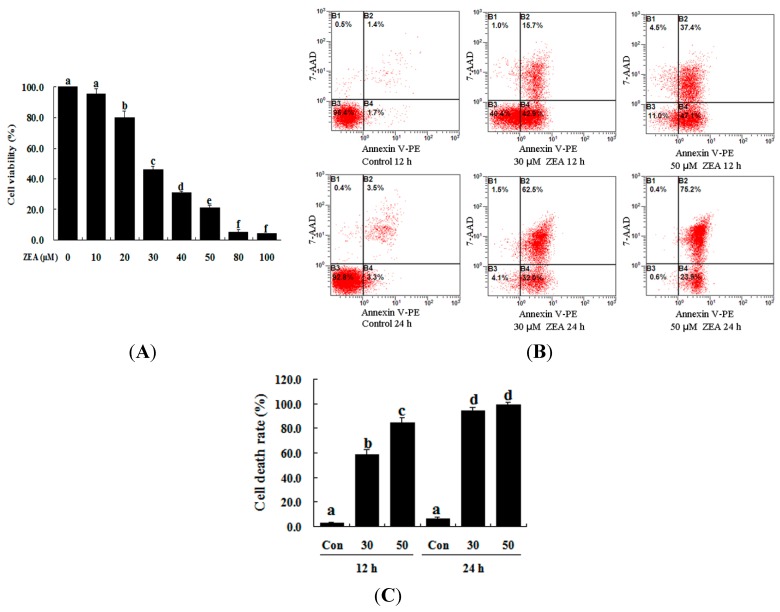

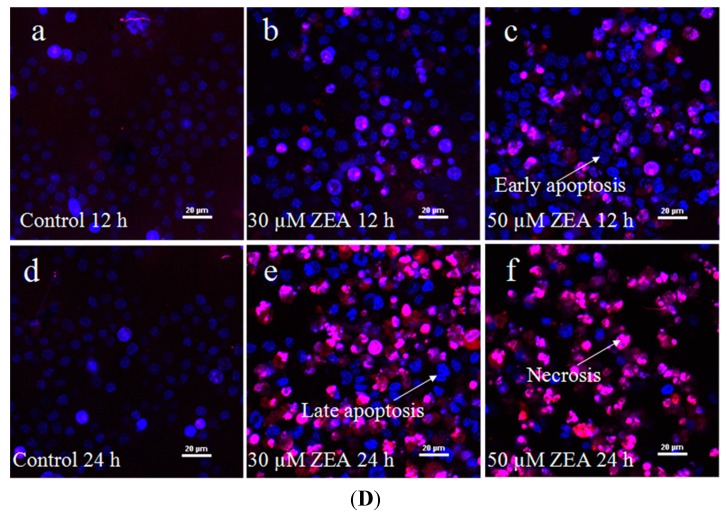
ZEA induces cell death in RAW 264.7 macrophages. (**A**) ZEA reduced the viability of RAW 264.7 macrophages in a dose-dependent manner. Cells were treated with 0, 10, 20, 30, 40, 50, 80 and 100 µM ZEA for 24 h and then processed for the MTT assay; (**B**,**C**) Apoptosis was detected via flow cytometry. B1 is the part of cell death caused by mechanical damage, B2 is the part of late apoptotic or necrotic cells, B3 is the part of the normal cells, and B4 is the part of early apoptotic cells. After exposure to 0, 30, and 50 µM ZEA for 24 h, RAW 264.7 macrophages were collected for Annexin V-PE/7-AAD staining followed by flow cytometric analysis. The statistical analysis is shown in the bar graphs. Data are presented as the mean ± SEM of three independent experiments. Bars with different letters are significantly different (*p* < 0.05); and (**D**) Representative photomicrographs of RAW 264.7 macrophages stained with Hoechst 33342 and PI fluorescent dye after exposure of the cells to 0 µM ZEA for 12 h (**a**); 30 µM ZEA for 12 h (**b**); 50 µM ZEA for 12 h (**c**); 0 µM ZEA for 24 h (**d**); 30 µM ZEA for 24 h (**e**); and 50 µM ZEA for 24 h (**f**). Apoptotic cells were characterized as having condensed or fragmented nuclei that stained deep blue, while necrotic cells were stained deep blue and deep red. Bar = 20 µm.

The toxic effect of ZEA on RAW 264.7 macrophages was examined by treatment with 0–100 µM ZEA for 24 h. Then the effect of this treatment on their proliferation was assessed using the 3-[4,5-dimethylthiazol-2-yl]-2,5-diphenyltetrazolium bromide assay (MTT) assay. ZEA was found to have very little effect at low doses (10 µM), while 20 µM ZEA significantly inhibited the growth of cells (Figure 1A, *p* < 0.05). These results showed that ZEA treatment clearly inhibited cell viability in a dose-dependent manner. The results of flow cytometry analysis also revealed that the apoptotic percentage of RAW 264.7 macrophages was significantly increased by ZEA treatment in a time-dependent manner compared with the control (Figure 1B,C, *p* < 0.05). The results of the Hoechst 33342 and propidium iodide (PI) staining assays showed that the early apoptotic cells were mainly observed after exposure to 30 and 50 µM ZEA for 12 h, but late apoptotic and necrotic cells were mainly observed after exposure to 30 and 50 µM ZEA for 24 h (Figure 1D).

### 2.2. Effect of ZEA on ER Stress in RAW 264.7 Macrophages

**Figure 2:**
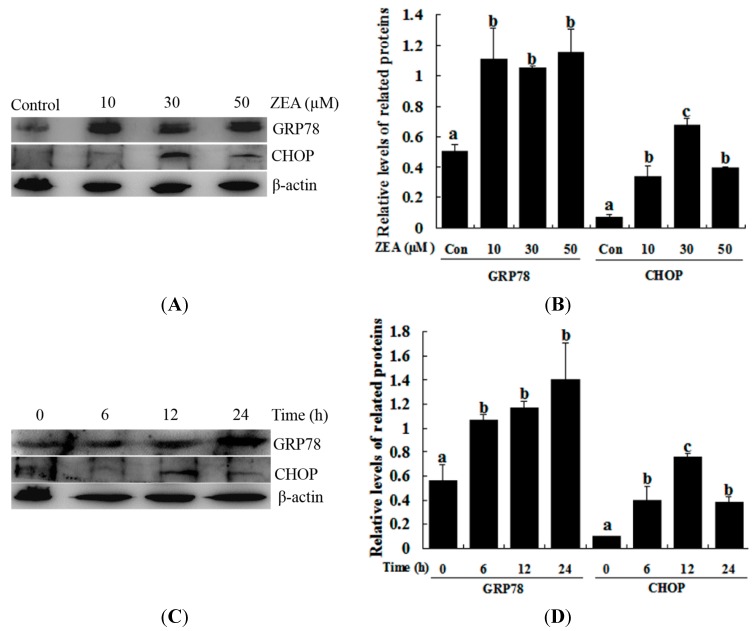
ZEA induces the ER stress-related proteins GRP78 and CHOP in RAW 264.7 macrophages. (**A**,**B**) The expression of GRP78 and CHOP was analyzed by western blot. Cells were treated with different concentrations of ZEA (control, 10, 30 and 50 µM) for 12 h; (**C**,**D**) the expression of GRP78 and CHOP was analyzed by western blot. RAW264.7 macrophages were treated for different times (0, 6, 12 and 24 h) with 30 µM ZEA. Analyses of the band intensity on the films are presented as the relative ratio of GRP78 and CHOP to β-actin. Statistical analysis is shown in the bar graphs. Data are presented as the mean ± SEM of three independent experiments. Bars with different letters are significantly different (*p* < 0.05).

The effect of ZEA on the ER stress-related gene (*GRP78* and *CHOP*) expression was studied via western blot analysis. ZEA treatment significantly induced the expression of GRP78 compared with the control, although treatment with 10, 30 and 50 µM ZEA and 30 µM ZEA for 6, 12 and 24 h did not produce significantly different results (Figure 2). CHOP protein was weakly detected after treatment with 10 µM ZEA for 12 h and 30 µM for 6 h, while treatment with 30 µM ZEA for 12 h dramatically induced the expression of CHOP, while treatment with 50 µM ZEA for 12 h and 30 µM for 24 h decreased the level of CHOP (Figure 2).

### 2.3. Effect of 4-PBA on ZEA-Induced Apoptosis in RAW 264.7 Macrophages

**Figure 3:**
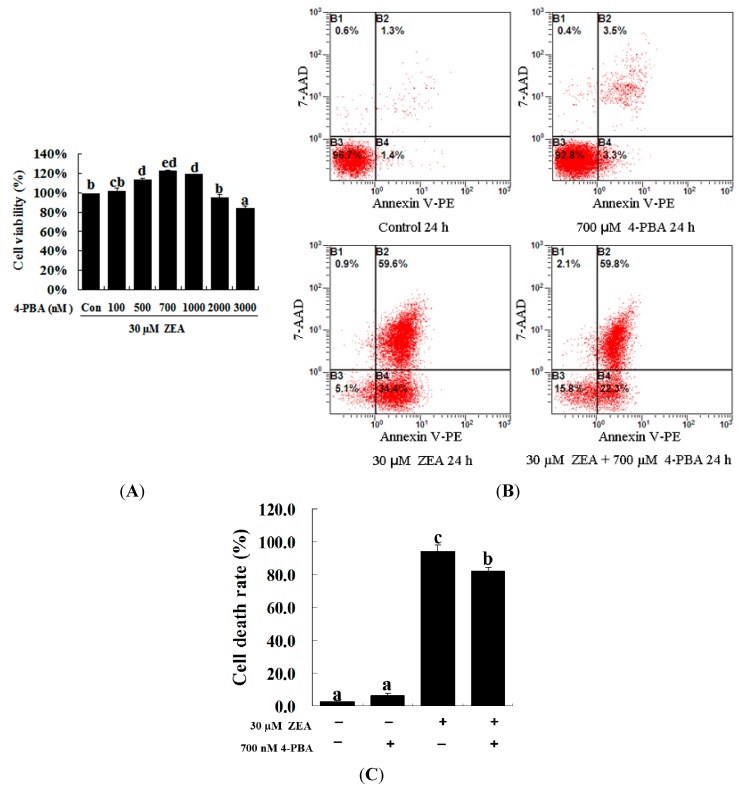
Effect of 4-PBA on the growth of ZEA-treated RAW 264.7 macrophages. (**A**) RAW 264.7 macrophages were treated with 30 µM ZEA in the presence or absence of 4-PBA for 24 h. Different doses of 4-PBA (0–3000 nM) were used to assess concentration effects on cell viability, and cells were then processed for the MTT assay; (**B**,**C**) Apoptosis analysis was detected via flow cytometry. After exposure to 30 µM ZEA in the presence or absence of 700 nM 4-PBA for 24 h, RAW 264.7 macrophages were collected for Annexin V-PE/7-AAD staining followed by flow cytometric analysis. Statistical analysis of the cell death is shown in the bar graphs. Data are presented as the mean ± SEM of three independent experiments. Bars with different letters are significantly different (*p* < 0.05).

**Figure 4:**
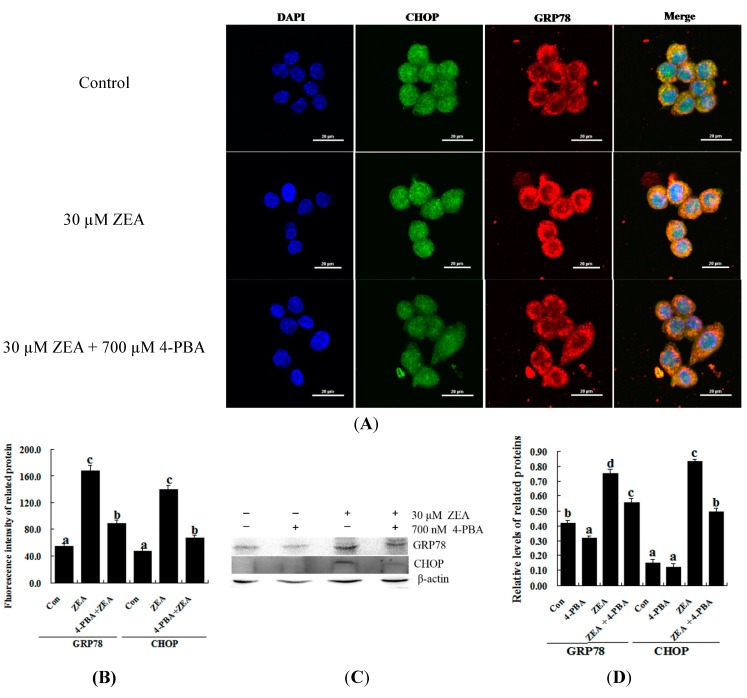
Effect of 4-PBA on the levels of GRP78 and CHOP in ZEA-treated RAW 264.7 macrophages. RAW 264.7 macrophages were treated 24 h with 30 µM ZEA or 30 µM ZEA with 700 nM 4-PBA. (**A**,**B**) Confocal immunofluorescence photomicrography showed the expression of CHOP protein (green fluorescence) and GRP78 protein (red fluorescence) in the control (the **upper** panels), ZEA (the **center** panels) or ZEA + 4-PBA cells (the **bottom** panels). Bars = 20 µm; (**C**,**D**) Western blot analysis of GRP78 and CHOP in ZEA-treated RAW 264.7 macrophages. The analyses of the band intensities on films are presented as the relative ratio of GRP78 and CHOP to β-actin. Statistical analysis is shown in the bar graphs. Data are presented as the mean ± SEM of three independent experiments. Bars with different letters are significantly different (*p* < 0.05).

To confirm the role of ER stress in ZEA-induced apoptosis, RAW 264.7 macrophages were treated with ZEA in the presence or absence of the ER stress inhibitor 4-PBA. Similar results were observed using the MTT assay and flow cytometry. Treatment with 700 nM 4-PBA significantly promoted cell viability and prevented cell apoptosis caused by 30 µM ZEA at 24 h, respectively (Figure 3A–C, *p* < 0.05). Meanwhile, 4-PBA markedly reduced the immunofluorescence staining of GRP78 and CHOP produced by 30 µM ZEA in RAW 264.7 macrophages (Figure 4A,B). In addition, Western blot analyses also revealed that the protein levels of GRP78 and CHOP in the ZEA-induced RAW 264.7 macrophages were significantly decreased after treatment of 4-PBA (Figure 4C,D, *p* < 0.05).

### 2.4. Attenuation of ZEA-Induced Cell Apoptosis by Suppression of CHOP Expression

**Figure 5:**
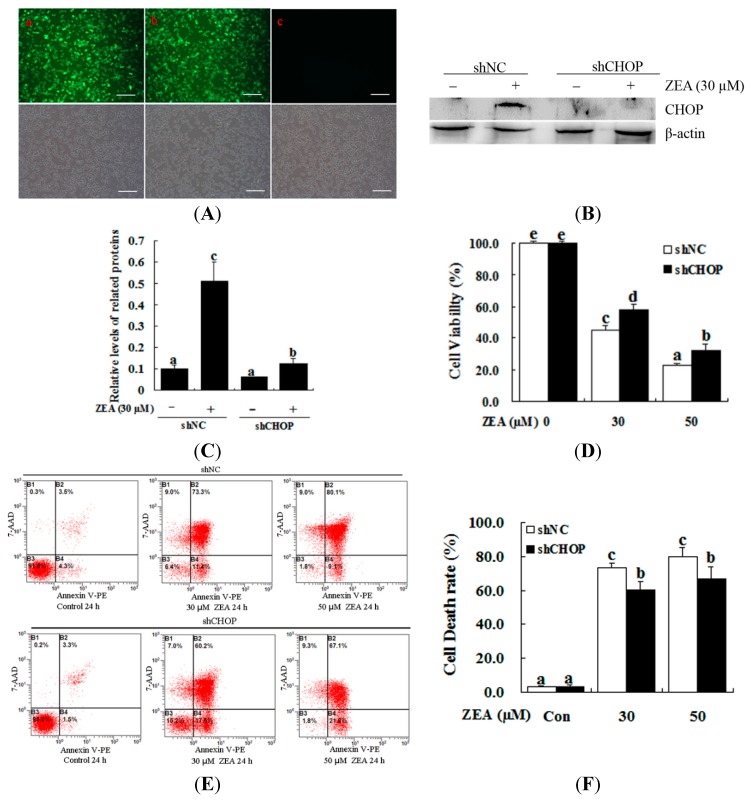
Effect of CHOP on the ZEA-induced cell death of RAW 264.7 macrophages. RAW 264.7 macrophages were transduced with *CHOP* or non-targeting lentiviral-mediated shRNAs. (**A**) The constructs themselves could express GFP, and the proportion of cells that were transduced was 90% by flow cytometry (data not shown). Fluorescence images of RAW 264.7 macrophages transducted for 48 h with negative control shRNA (shNC) (**a**), shCHOP (**b**) and control (**c**) virus-containing supernatant; GFP expression was observed under light (**top** panels) or fluorescence microscopy (**bottom** panels). Bars = 50 µm; (**B**,**C**) The expression levels of CHOP were determined by western blot analysis. RAW 264.7 macrophages were transduced with lentiviruses expressing either shCHOP or shNC, and were then treated with 30 μM ZEA for 24 h. The analyses of the band intensity on the films are presented as the relative ratio of CHOP to β-actin. Statistical analysis is shown in the bar graphs; (**D**) Transduced RAW 264.7 macrophages with lentiviruses expressing either shCHOP or shNC were treated with 0, 30 and 50 μM ZEA and then cell viability was measured after 24 h by the MTT assay; (**E**,**F**) RAW 264.7 macrophages were tranduced with shNC or shCHOP to confirm the effects of shCHOP. To determine the apoptotic effect of ZEA on the RAW 264.7 macrophages, cells were transduced with shNC or shCHOP for 48 h and then incubated for 24 h in the absence or presence of 30 μM ZEA. Apoptosis was determined by Annexin V-PE/7-AAD staining. B1 is the part of cell death caused by mechanical damage, B2 is the part of late apoptotic or necrotic cells, B3 is the part of the normal cells, and B4 is the part of early apoptotic cells. Statistical analysis is shown in the bar graphs. Data are presented as the mean ± SEM of three independent experiments. Bars with different letters are significantly different (*p* < 0.05).

To further examine the role of ER stress in ZEA-induced cellular apoptosis, we used lentiviral-transduced shRNA to down-regulate *CHOP* expression. With this strategy, we confirmed that the ER stress pathway is required for ZEA-induced apoptosis in RAW 264.7 macrophages (Figure 5A–C). The MTT assay showed that *CHOP* knockdown effectively increased the cell viability compared with the control after treatment with 30 µM ZEA for 24 h (Figure 5D, *p* < 0.05). Treatment with 30 µM ZEA for 24 h after transducing the shCHOP lentivirus significantly suppressed ZEA-induced cell apoptosis compared with the control (Figure 5E,F, *p* < 0.05).

## 3. Discussion

Although numerous studies have demonstrated that ZEA induces reproductive disorders and is immunotoxic, hepatotoxic and genotoxic in both humans and animals, the detailed mechanisms of ZEA toxicity have not been fully elucidated. Many mechanisms have been proposed to explain how ZEA functions as a toxic agent in many signaling pathways. Previous research has demonstrated that RAW 264.7 macrophages could be induced to undergo cell death, primarily through necrosis, with exposure to 50 µM ZEA for 24 h [20]. Our partial results were consistent with the findings of previous reports. We discovered that early apoptotic cells were mainly observed after exposure to 30 and 50 µM ZEA for 12 h, but after exposure for 24 h, late apoptotic and necrotic cells were mainly observed. The present findings support the idea that ZEA treatment significantly reduces the viability of RAW 264.7 macrophages and induces cell death in a dose- and time-dependent manner. However, cell apoptosis may be mainly caused by ZEA treatment at low doses or for a short time, while both apoptosis and necrosis may result from ZEA treatment at high doses and for a long time.

In previous studies, the mitochondrion-initiated intrinsic pathway has been found to be involved in the process of ZEA-mediated apoptosis. ZEA treatment induces the formation of DNA adducts and ladders and leads to micronuclei and chromosomal aberrations [31,32]. This process also results in the inhibition of DNA and protein synthesis, glutathione depletion, stress protein induction, and oxidative stress-mediated cell death [17,33,34]. ZEA not only induces apoptosis in human hepatocytes but also results in apoptotic cell death in porcine granulosa cells via the caspase-dependent, mitochondrial signaling pathway [14,18,35]. ZEA induces cell death by regulating Bcl-2 family proteins, which can downregulate Bcl-2 and upregulate Bax expression [29,36]. Cell death caused by ZEA is closely related to its potential to cause DNA damage and ROS generation in RAW 264.7 macrophages in which the activation of p53 and MAPK plays a critical role. Our study shows that ZEA induces apoptosis via ER stress in mouse Leydig cells [30]; in summary, the resulting outcomes are believed to differ according to the types of cells examined and the mycotoxins exposed.

Previous studies have reported that ER stress triggers cell death via an alternative intrinsic apoptotic pathway that does not require cytochrome c or Apaf-1 [37]. The accumulation of unfolded or misfolded proteins in the ER can cause ER stress. ER transmembrane proteins dissociate with the ER chaperone molecular GRP78, which binds to the accumulating unfolded proteins and transduces the ER stress signaling pathways. This signaling pathway up-regulates the expression of molecular chaperones in the ER, subsequently restoring homeostasis and promoting cell survival. However, with severe and prolonged ER stress, the pro-apoptotic factor CHOP is induced and causes cell apoptosis in some cell types [38,39,40]. CHOP exists downstream of all of these ER stress signaling pathways. CHOP regulates the expression of a set of stress-induced genes involved in cell death. CHOP down-regulates the expression of Bcl-2 and exaggerates the production of ROS [41]. Furthermore, CHOP induces cell apoptosis in RAW 264.7 macrophages through the translocation of a pro-apoptotic protein Bax from the cytosol to the mitochondria [42]. Previous studies have demonstrated that nitric oxide (NO) induces ER stress and apoptosis via the ATF6/CHOP signaling pathway in RAW 264.7 macrophages [43]. In this study, the related marker molecules of ER stress, GRP78 and CHOP, were found to be up-regulated in the ZEA-treated cells with marked dose- and time-dependent increases. Furthermore, we also found that 4-PBA (an ER stress inhibitor) markedly reduced the ZEA-induced up-regulation of GRP78 and CHOP in RAW 264.7 macrophages and subsequently increased cell death. Furthermore, the knockdown of *CHOP* with lentivirus-mediated shRNA diminished ZEA-induced cell death. Taken together, these results suggest that ZEA treatment might activate the ER stress response, and that *CHOP* might play an important role in the regulation of cell death in RAW 264.7 macrophages.

## 4. Materials and Methods

### 4.1. Materials

ZEA and 4-phenylbutyrate (4-PBA) were purchased from Sigma Chemical Co. (St. Louis, MO, USA). Dulbecco’s modified Eagle’s medium (DMEM) and fetal bovine serum (FBS) were purchased from Gibco (Grand Island, NY, USA). An Annexin V-PE/7-AAD kit, Total Protein Extraction Kit and BCA Protein Assay Kit were purchased from Nanjing Keygen Biotech Co., Ltd. (Nanjing, Jiangsu, China). The Apoptosis and Necrosis Assay Kit was purchased from Beyotime Institute of Biotechnology (Shanghai, China). Anti-β-actin antibody was obtained from Beijing CWBIO Co., Ltd. (Beijing, China). Anti-GRP78 and anti-CHOP antibodies were obtained from Santa Cruz Biotechnology (Santa Cruz, CA, USA).

### 4.2. Cell Culture and Treatment

RAW 264.7 macrophages were cultured in DMEM supplemented with 10% FBS and 100 IU/mL penicillin and 100 μg/mL streptomycin solution in a humidified incubator at 37 °C and 5% CO_2_. The growth medium was refreshed every 2–3 days. When the cells reached 70%–80% confluence, they were exposed to various concentrations (0–100 μM) of ZEA. At various times (0–48 h) during the treatment, cells were collected and processed for further experiments. In another experiment, RAW 264.7 macrophages were exposed to 30 and 50 µM of ZEA in the presence or absence of the ER stress inhibitor 4-PBA. After 24 h of culture, the cells were collected for flow cytometry and western blotting analysis.

### 4.3. Measurement of Cell Viability

To test the effects of ZEA on the changes in cell viability, cells were seeded into a 96-well plate at 5 × 10^3^ cells/well. After culture for 24 h, the cells were treated with various concentrations of ZEA from 0 to 100 µM for 24 h. Cells were treated with 0.5 mg/mL MTT after exposure to ZEA and were then incubated for an additional 4 h. At the end of the experiments, the cell growth medium was replaced by 150 µL DMSO and colorimetric measurements were performed with an ELISA plate reader at 570 nm using a microplate reader (Model 680, Bio-Rad, Hercules, CA, USA).

### 4.4. Apoptosis Analysis with Annexin V-PE/7-AAD Staining

After 24 h of ZEA treatment, apoptotic cells were quantified with an Annexin V-PE and 7-AAD apoptosis detection kit. Cells were washed twice with cold PBS by centrifugation, and their density was adjusted to 1 × 10^5^ cells/mL. Cells were then resuspended in 50 μL binding buffer, after which, 5 μL 7-AAD was added and the mixture was incubated for 15 min. Then, 450 μL binding buffer was added, followed by addition of 1 μL Annexin V-PE and incubation for 15 min. Detection by flow cytometry (EPICS Altra, Beckman Coulter Cytomics Altra, Brea, CA, USA) was conducted within 1 h. The number of early-apoptotic cells was determined by counting the percentage of Annexin V-PE^+^/7-AAD^−^ cells (“^+^” means that the cell could be stained by Annexin V-PE or 7-AAD; “^−^” means that the cell could not be stained by Annexin V-PE or 7-AAD). Late apoptotic or necrotic cells were obtained by counting the percentage of Annexin V-PE^+^/7-AAD^+^ cells. Annexin V-PE^−^/7-AAD^−^ cells were considered to be surviving cells.

### 4.5. Examination of ZEA-Induced Cell Death by Hoechst-PI Staining

To assess the apoptosis or necrosis phenomenon, Hoechst 33342 and PI staining assays were carried out. For these, 1 × 10^5^ RAW 264.7 macrophages per well were cultured over night in 24-well plates and treated with 0, 30 and 50 μM ZEA for 12 and 24 h. The resulting cell morphology was assessed using an Apoptosis and Necrosis Assay Kit (Beyotime Institute of Biotechnology, Shanghai, China). Briefly, cells in 24-well plates were trypsinized and washed twice with PBS. The cells were then stained with Hoechst 33342 and PI for 30 min at 4 °C in the dark. Apoptotic and necrotic cells were observed using a Nikon epifluorescence microscope (Nikon Eclipse 80i; Nikon, Tokyo, Japan).

### 4.6. Immunofluorescence Staining

RAW 264.7 macrophages were cultured in 24-well plates and treated with 30 µM of ZEA in the presence or absence of 700 nM 4-PBA for 24 h. Immunofluorescence staining of GRP78 and CHOP was performed. RAW 264.7 macrophages were first fixed in 4% paraformaldehyde for 20 min, and then permeabilized with 0.1% Triton X-100 in PBS for 20 min. They were subsequently blocked with 5% BSA in PBS for 1 h at room temperature. The membranes were then incubated with the chosen antibodies, including antibodies to CHOP and GRP78, at a dilution of 1:50 in TBST for 2 h at 37 °C. After washing followed by incubation with anti-rabbit secondary antibody (Invitrogen, A21206; 1:500 dilution) and anti-goat secondary antibody (Invitrogen, A21432; 1:500 dilution) for 1 h at 37 °C, the nuclei were stained with 4′,6-diamidino-2-phenylindole (DAPI) for 5 min. The fluorescent signals were examined under a Nikon epifluorescence microscope (Nikon Eclipse 80i, Nikon, Tokyo, Japan).

### 4.7. Transducing Short Hairpin Interfering RNAs (shRNAs) via Lentiviral Infection

Recombinant lentiviral vectors encoding *CHOP* shRNA and shNC were constructed in our laboratory. ShNC contains a scrambled sequence, which does not have any effect on the expression of any gene. The recombinant lentivirus vector was packaged and transfected into HEK 293T cells. The medium was harvested 48 h after transfection, purified by low-speed centrifugation, and filtered through a 0.45-µm polyvinylidene difluoride (PVDF) filter. RAW 264.7 macrophages were cultured in DMEM with 10% FBS. An appropriate amount of virus (*MOI* = 20) was diluted in the culture medium (containing 8 µg/mL of polybrene) on the next day. ShCHOP and shNC viral supernatant were added. The following day, the medium containing the virus was removed and replaced with fresh culture medium. Following transduction (48 h), the medium was then replaced without or with 30 µM of ZEA for another 24 h.

### 4.8. Western Blot Analysis

After treatment, the cells were washed with cold PBS and lysed on ice for 30 min in radioimmunoprecipitation (RIPA) buffer. After centrifugation at 14,000 rpm for 15 min at 4 °C, the supernatant was then transferred to a new tube and the protein concentration was determined by the BCA assay. The total cellular protein of 50 μg was electrophoresed on a 12% sodium dodecyl sulfate polyacrylamide gradient (SDS-PAGE) gel and electro-transferred onto PVDF membranes. The membranes were then blocked with 5% fatty acid-free milk in Tris-buffered saline (TBS) containing 0.5% Tween-X-100 for 1 h at room temperature and incubated overnight at 4 °C in blocking solution containing anti-GRP78 and anti-CHOP primary antibodies. The next day, the membranes were washed with TBST and incubated with secondary antibody conjugated to horseradish peroxidase (1:5000; Zhongshan Golden Bridge Biotechnology, Nanjing, China) at room temperature for 1 h. Finally, the immunoreactive bands were visualized using the Gel Image System (Tannon, Biotech, and Shanghai, China) and then digitized with the Quantity One Software (Bio-Rad).

### 4.9. Statistical Analysis

All experiments were replicated at least three times for each group, and the experimental results are presented as the mean ± SEM. Data were analyzed with one-way ANOVA, followed by Fisher’s least significant different test (Fisher LSD) and Independent-Samples T test with SPSS (Statistical Package for the Social Sciences) software (Version 13.0; SPSS, Inc., Chicago, IL, USA). Differences were considered significant when *p* < 0.05.

## 5. Conclusions

In summary, this study demonstrates that ER stress is involved in the ZEA-induced cell death of RAW 264.7 macrophages in which the activation of *CHOP* plays a critical role. This study offers new insight into the molecular mechanisms of ZEA toxicity in macrophages.
